# Protein arginine methyltransferases in cardiovascular disease: mechanisms, therapeutic potential, and future directions

**DOI:** 10.3389/fphys.2026.1904070

**Published:** 2026-09-01

**Authors:** Ting Zhang, Hong Lei, Chunmei Zhang

**Affiliations:** Department of Cardiovascular Medicine, Karamay Central Hospital, Karamay, Xinjiang, China

**Keywords:** ADMA, cardiovascular diseases, epigenetic regulation, protein arginine methyltransferases, therapeutic targets

## Abstract

Protein arginine methyltransferases (PRMTs) constitute a family of nine enzymes that catalyze the post-translational methylation of arginine residues on histone and non-histone proteins. This epigenetic modification regulates diverse cellular processes including gene transcription, RNA splicing, signal transduction, and protein-protein interactions. Emerging evidence indicates that dysregulated PRMT activity contributes significantly to the pathogenesis of cardiovascular diseases (CVDs), including atherosclerosis, hypertension, heart failure, and myocardial infarction. This review synthesizes current literature on PRMT-mediated mechanisms in cardiovascular pathology, examining the roles of individual PRMT isoforms, their downstream substrates, and the therapeutic implications of PRMT modulation. PRMT1, PRMT5, and PRMT7 demonstrate cardioprotective functions under physiological conditions but contribute to pathological remodeling when dysregulated. The PRMT-DDAH-ADMA axis emerges as a critical regulator of endothelial nitric oxide pro-duction and vascular homeostasis. Type I PRMTs generate asymmetric dimethylarginine (ADMA), an endogenous nitric oxide synthase inhibitor that promotes endothelial dysfunction and atherosclerosis. Type II PRMTs, particularly PRMT5, regulate cardiac fibroblast activation, hypertrophic signaling, and vascular smooth muscle cell phenotypic switching. PRMTs represent promising therapeutic targets for cardiovascular disease intervention. Selective PRMT inhibitors have demonstrated efficacy in preclinical models of cardiac hypertrophy, fibrosis, and ischemic injury. Future research should focus on developing isoform-specific inhibitors with favorable safety profiles and establishing biomarker-guided patient selection strategies for PRMT-targeted therapies.

## Introduction

Cardiovascular diseases remain the leading cause of morbidity and mortality worldwide, accounting for approximately 10.8 million deaths annually (Di Cesare et al., 2024). Despite advances in prevention and treatment, the global burden of cardiovascular disease continues to rise, driven by aging populations and the increasing prevalence of metabolic disorders. This persistent challenge underscores the urgent need to identify novel molecular mechanisms and therapeutic targets that can complement existing treatment paradigms (Zhu, 2026).

Protein arginine methylation, catalyzed by the protein arginine methyltransferase (PRMT) family of enzymes, has emerged as a critical post-translational modification with far-reaching implications for cardiovascular biology (Couto et al., 2020). The PRMT family comprises nine members (PRMT1-9) that catalyze the transfer of methyl groups from S-adenosylmethionine (SAM) to the guanidino nitrogen atoms of arginine residues (Pahlich et al., 2006). This modification can produce three distinct products: omega-N-monomethylarginine (MMA), asymmetric dimethylarginine (ADMA), and symmetric dimethylarginine (SDMA) (Tan et al., 2021). Type I PRMTs (PRMT1, 2, 3, 4, 6, and 8) generate ADMA, while Type II PRMTs (PRMT5 and 9) produce SDMA (Gui et al., 2014; Yang et al., 2015).

The biological significance of PRMT-mediated arginine methylation extends beyond epigenetic regulation. The byproduct ADMA functions as an endogenous competitive inhibitor of nitric oxide synthase (NOS), directly linking PRMT activity to vascular homeostasis (Anthony et al., 2005; Pope et al., 2009). Elevated plasma ADMA concentrations have been consistently associated with endothelial dysfunction, atherosclerosis, hypertension, and adverse cardiovascular outcomes (Dowsett et al., 2020). Furthermore, PRMTs regulate key cardiovascular signaling pathways through methylation of non-histone proteins including transcription factors, signaling molecules, and ion channels (Tang et al., 2025).

Recent studies have revealed cell-type-specific roles for individual PRMT isoforms in cardiovascular pathology. Cardiac-specific PRMT1 ablation causes heart failure through dysregulation of CaMKII activity (Pyun et al., 2018), while PRMT5 mediates cardiac fibroblast activation and fibrotic remodeling (Dong et al., 2023). PRMT7 demonstrates cardioprotective effects through regulation of JAK/STAT signaling and oxidative stress responses.These protective actions appear to stem from PRMT7’s regulation of stress-response and survival pathways rather than from its MMA-restricted catalytic profile, highlighting pathway-specific functions that distinguish PRMT7 from other PRMT isoforms (Tran et al., 2025). PRMT expression and activity are also influenced by upstream regulatory cues, including inflammatory cytokines, metabolic and oxidative stress, and lipid-responsive transcriptional pathways such as SREBP, which modulate DDAH expression and methylarginine metabolism (Kim et al., 2015; Sun et al., 2015). These findings establish PRMTs as both therapeutic targets and potential biomarkers for cardiovascular disease.

While previous reviews have summarized the general involvement of PRMTs in cardiovascular diseases, the present manuscript provides a more mechanistically focused and cell-type–specific analysis of PRMT isoforms, along with an expanded discussion of therapeutic targeting strategies.

### PRMT family: classification and structure

The protein arginine methyltransferase family consists of nine evolutionarily conserved enzymes that share a common catalytic domain structure while exhibiting distinct substrate specificities and cellular functions (Tewary et al., 2019).All PRMTs contain a conserved methyltransferase domain that facilitates SAM binding and methyl group transfer, with additional domains mediating substrate recognition and protein-protein interactions (Rust et al., 2011).

PRMT1, the predominant type I enzyme, accounts for approximately 85% of total cellular arginine methyltransferase activity (Nicholson et al., 2009). It methylates a broad spectrum of substrates including histones, RNA-binding proteins, and transcription factors. The crystal structure of PRMT1 reveals a dimeric arrangement essential for catalytic activity, with the active site positioned at the dimer interface (Nicholson et al., 2009).PRMT4 (also known as CARM1) functions as a transcriptional coactivator with distinct substrate preferences, including histone H3 arginine 17 (Suresh et al., 2021).

Type II PRMTs, exemplified by PRMT5, form oligomeric complexes with essential cofactors MEP50 and pICln for optimal activity (Ho et al., 2013). PRMT5 symmetrically dimethylates arginine residues on histones and non-histone proteins, generating transcriptional repressive marks while also regulating spliceosome assembly and mRNA processing (Bezzi et al., 2013). PRMT9, the second type II enzyme, exhibits more restricted substrate specificity and remains less characterized in cardiovascular contexts (Yang et al., 2015).

### Arginine methylation products and metabolism

The methylation products generated by PRMT activity have distinct biological fates and functional consequences. MMA serves as an intermediate in the dimethylation pathway and may have independent signaling functions (Thiebaut et al., 2021). ADMA, produced exclusively by type I PRMTs, accumulates in tissues and plasma through proteolysis of methylated proteins. As a structural analog of L-arginine, ADMA competitively inhibits all three NOS isoforms, with particular potency against endothelial NOS (eNOS) (Pope et al., 2009).

**Figure 1:**
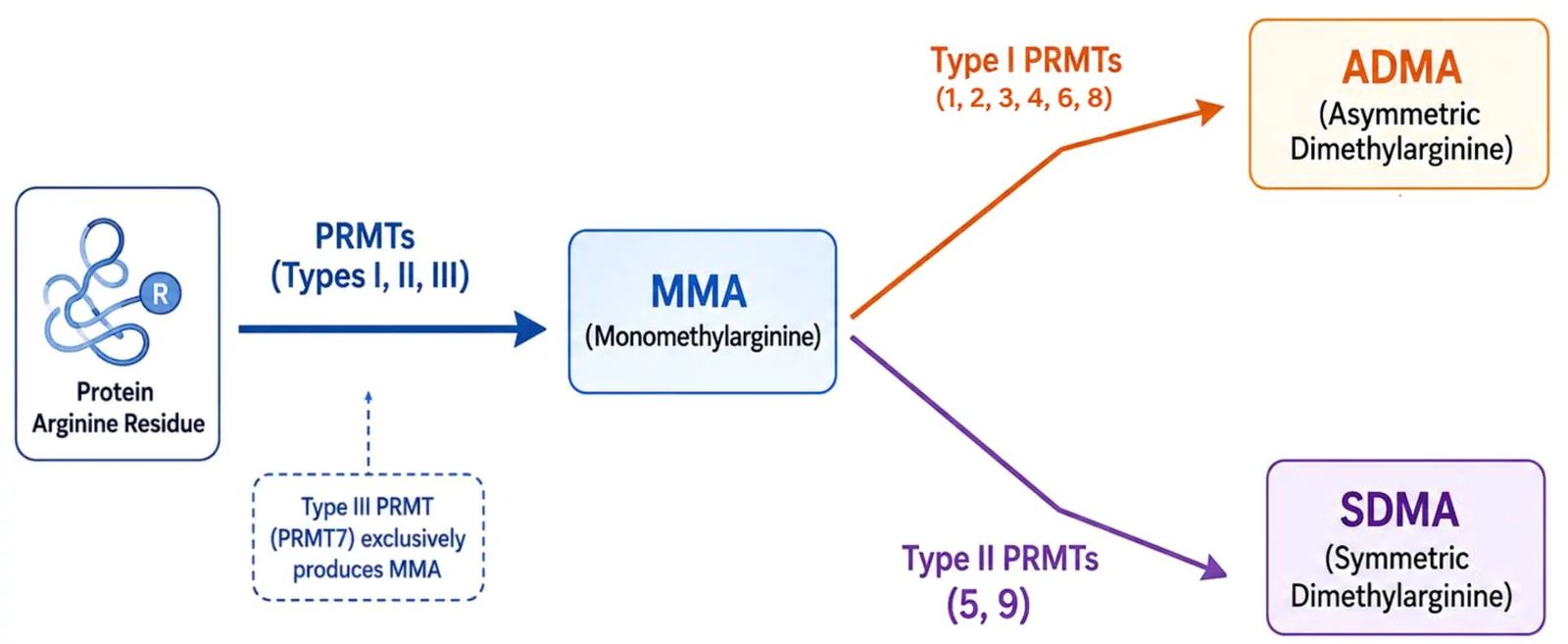
Schematic representation of protein arginine methylation pathways and PRMT isoform specificity. Free protein arginine residues undergo initial monomethylation to form monomethylarginine (MMA) by all types of protein arginine methyltransferases (PRMTs), utilizing S-adenosylmethionine (SAM) as the methyl donor and generating S-adenosylhomocysteine (SAH) as a byproduct. MMA serves as a transient intermediate that can be sequentially modified. Type I PRMTs (PRMT1, 2, 3, 4, 6, and 8) catalyze subsequent asymmetric dimethylation to produce asymmetric dimethylarginine (ADMA), whereas Type II PRMTs (PRMT5 and 9) catalyze symmetric dimethylation to form symmetric dimethylarginine (SDMA). In contrast, Type III PRMT (PRMT7) functions exclusively to generate MMA as its final product and does not catalyze further dimethylation reactions.

SDMA, generated by type II PRMTs, does not directly inhibit NOS but has been associated with adverse cardiovascular outcomes through mechanisms involving competition with L-arginine for cellular uptake and effects on vascular inflammation (Kahraman et al., 2017) (**Figure 1**). The biological half-life of free methylarginines is regulated by dimethylarginine dimethylaminohydrolases (DDAH-1 and DDAH-2), which hydrolyze ADMA and SDMA to citrulline and dimethylamine (Kozlova et al., 2022).

The PRMT-DDAH-ADMA axis represents a critical regulatory network governing nitric oxide bioavailability and endothelial function (Pope et al., 2009). Dysregulation at any level of this path-way can result in pathological ADMA accumulation, endothelial dysfunction, and accelerated atherosclerosis (Leiper, 2005).

Cardiovascular tissues exhibit distinct patterns of PRMT expression that reflect their special-ized functions. In the heart, PRMT1 is highly expressed in cardiomyocytes and demonstrates dynamic regulation during development and stress responses (Jeong et al., 2019). Cardiac PRMT1 expression increases under hypertrophic stimuli but decreases in failing hearts (Zhang et al., 2024). PRMT5 is expressed in cardiomyocytes, cardiac fibroblasts, and endothelial cells, with elevated levels observed in pathological hypertrophy and heart failure (Katanasaka et al., 2025).

Vascular tissues show enriched expression of PRMT1 in endothelial cells, where they regulate angiogenesis, vascular permeability, and inflammatory responses (Katanasaka et al., 2025). Vascular smooth muscle cells (VSMCs) express PRMT5 at high levels, and this expression increases during phenotypic switching from contractile to synthetic states (Liu et al., 2022). PRMT7 is expressed in both endothelial cells and cardiomyocytes, where it maintains vascular homeostasis and protects against oxidative stress (Ahn et al., 2022).

### PRMT-mediated mechanisms in cardiovascular disease

Endothelial dysfunction represents an early and critical event in atherosclerosis and other car-diovascular diseases. The PRMT-DDAH-ADMA axis is centrally involved in the regulation of endothelial nitric oxide production and vascular homeostasis (Pope et al., 2009). Under physiological conditions, PRMT activity generates ADMA-containing proteins, which upon proteolysis release free ADMA into circulation. DDAH enzymes, particularly DDAH-2 expressed in vascular endothelium, efficiently metabolize ADMA to prevent accumulation (Leiper et al., 2007).

Pathological conditions including hypercholesterolemia, diabetes, hypertension, and inflammation disrupt this balance by upregulating PRMT expression and/or inhibiting DDAH activity (Böger et al., 2000). Oxidative stress plays a particularly important role, as hydrogen peroxide and other reactive oxygen species directly inhibit DDAH activity while potentially stimulating PRMT expression (Morales et al., 2015).

The resulting ADMA accumulation competitively inhibits eNOS, reducing nitric oxide bioavailability while potentially promoting eNOS uncoupling and superoxide generation (Vallance and Leiper, 2004).

**Figure 2:**
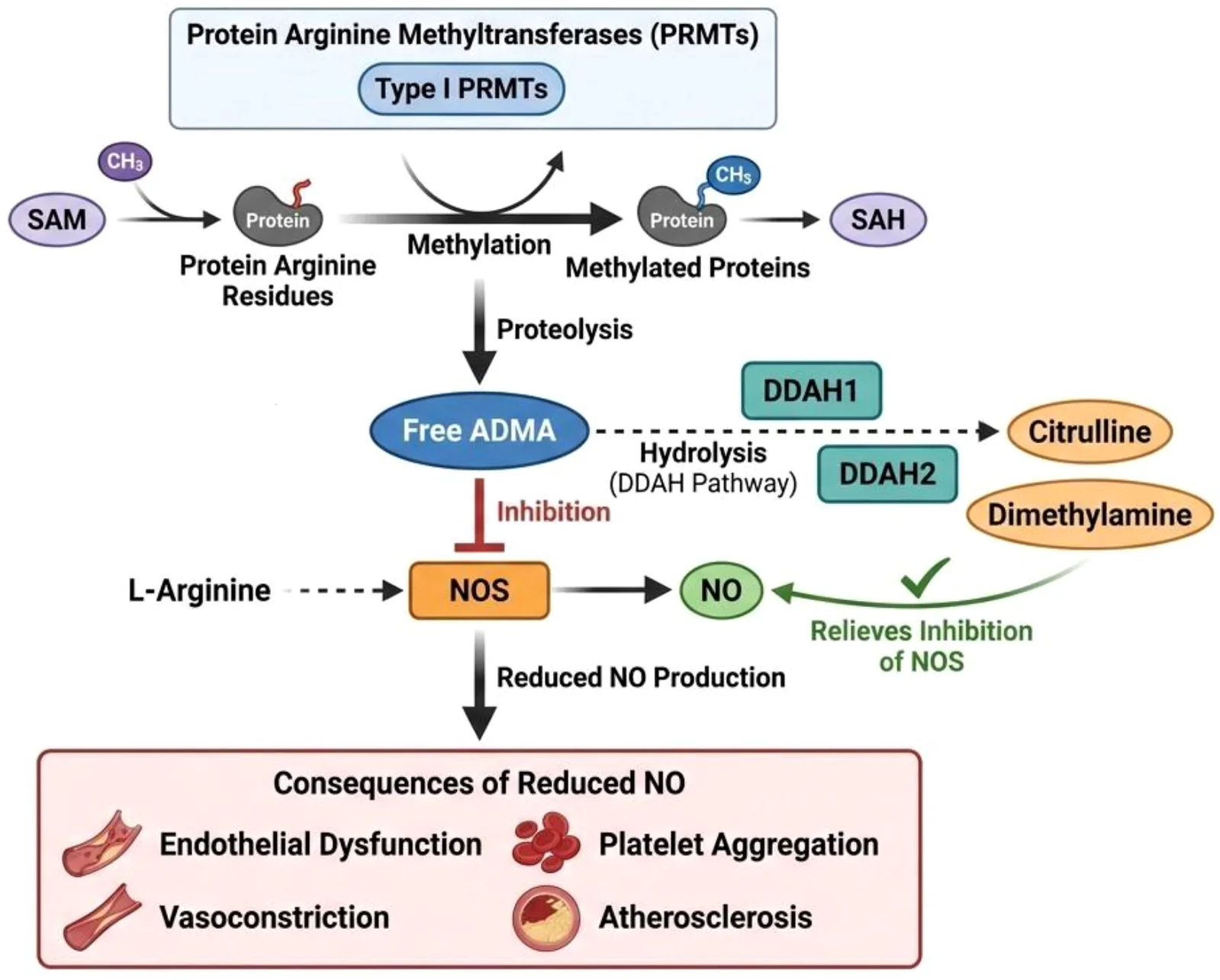
The PRMT-DDAH-ADMA axis in cardiovascular disease. Type I PRMTs methylate protein arginine residues using S-adenosylmethionine (SAM) as the methyl donor, producing methylated proteins and S-adenosylhomocysteine (SAH). Proteolysis of methylated proteins releases free asymmetric dimethylarginine (ADMA), which competitively inhibits nitric oxide synthase (NOS), thereby reducing NO production. DDAH1 and DDAH2 enzymes metabolize ADMA to citrulline and dimethylamine, thereby preventing NOS inhibition. Dysregulation of this pathway leads to endothelial dysfunction, vasoconstriction, platelet aggregation, and atherosclerosis.

Clinical studies have consistently demonstrated elevated plasma ADMA levels in patients with cardiovascular disease (Sibal et al., 2010). ADMA concentrations correlate with disease severity, and reductions in ADMA through therapeutic interventions are associated with improved vascular function (Maas, 2005). The prognostic value of ADMA has been validated in multiple cohorts, with elevated levels predicting cardiovascular events independent of traditional risk factors (Schulze et al., 2006) (**Figure 2**).

PRMT1 occupies a central position in cardiac biology due to its high expression levels and diverse substrate repertoire. Cardiac-specific PRMT1 ablation in mice results in progressive heart failure characterized by cardiac dilation, contractile dysfunction, and premature mortality (Pyun et al., 2018).Mechanistic studies revealed that PRMT1 methylates CaMKII at arginine residues 9 and 275, inhibiting its autophosphorylation and subsequent activation. Loss of PRMT1-mediated CaMKII methylation results in constitutive CaMKII activation, calcium mishandling, and cardiac dysfunction (Pyun et al., 2018).

PRMT1 also regulates alternative splicing in cardiomyocytes through methylation of splicing factors including SRSF1. During hypertrophic stress, PRMT1-mediated SRSF1 methylation modulates splicing of genes involved in calcium handling, contractility, and hypertrophic signaling. This epitranscriptomic regulation represents a novel mechanism through which PRMT1 maintains cardiac homeostasis (Yan et al., 2024).

Under ischemic conditions, PRMT1 expression decreases in the myocardium, contributing to increased susceptibility to ischemia-reperfusion injury. The decline in PRMT1 activity impairs cellular stress responses and increases cardiomyocyte apoptosis. Conversely, PRMT1 overexpression or pharmacological enhancement of PRMT1 activity demonstrates cardioprotective effects in preclinical models (Boovarahan and Kurian, 2022). Beyond stress-induced remodeling, PRMT1-dependent RNA processing is fundamentally required for basal cardiac survival and structural integrity. Evidence from conditional knockout models reveals that deleting PRMT1 specifically in juvenile cardiomyocytes precipitates aggressive dilated cardiomyopathy and early mortality in mice. At the molecular level, this phenotypic collapse is driven by widespread disruptions in transcriptional fidelity, characterized by the emergence of novel, cryptic alternative splicing variants of essential cardiac genes—most notably *Asb2*, *Fbxo40*, *Nrap*, and *Eif4a2*. Intriguingly, the aberrant splicing of *Eif4a2* yields distinct protein isoforms that undergo accelerated ubiquitination and premature proteasomal clearance, demonstrating that PRMT1 serves as an indispensable gatekeeper of both post-transcriptional accuracy and proteostatic equilibrium in the myocardium (Murata et al., 2018).

PRMT5 has emerged as a key regulator of cardiac fibroblast activation and fibrotic remodeling. Cardiac fibrosis, characterized by excessive accumulation of extracellular matrix proteins, contributes to diastolic dysfunction, arrhythmias, and progression to heart failure (Katanasaka et al., 2024). PRMT5 expression is upregulated in activated cardiac fibroblasts and mediates transforming growth factor-beta (TGF-*β*) signaling through methylation of Smad3 (Dong et al., 2023).

In adriamycin-induced cardiomyopathy, PRMT5 inhibition or fibroblast-specific PRMT5 knockout attenuates cardiac fibrosis and preserves left ventricular function (Dong et al., 2023; Katanasaka et al., 2024). PRMT5 promotes TGF-*β*-induced fibroblast activation through symmetric dimethylation of histone H4 arginine 3 (H4R3me2s) at fibrotic gene promoters, creating a repressive chromatin environment that paradoxically activates fibrotic gene expression through mechanisms involving interactions with other epigenetic modifiers (Dong et al., 2023).

In cardiomyocytes, PRMT5 demonstrates context-dependent effects on hypertrophic remodeling. Global PRMT5 deletion is embryonically lethal, while cardiac-specific overexpression of PRMT5 exacerbates pressure overload-induced hypertrophy and heart failure (Katanasaka et al., 2025). PRMT5 regulates hypertrophic gene expression through symmetric dimethylation of histones and non-histone proteins including transcription factors. However, other studies suggest PRMT5 may have protective effects under certain conditions, highlighting the complexity of PRMT5 biology in the heart (Katanasaka et al., 2025).

PRMT7 exhibits unique enzymatic properties as a type III PRMT that can only generate MMA and represents the only PRMT that acts as a monomer (Krause et al., 2007). In the cardiovascular system, PRMT7 demonstrates predominantly protective functions. Cardiac-specific PRMT7 ablation causes cardiac hypertrophy, fibrosis, and dysfunction through dysregulation of the Wnt/*β*-catenin signaling pathway (Ahn et al., 2022).

PRMT7 protects against oxidative stress and DNA damage in cardiomyocytes by regulating the expression of antioxidant genes and DNA repair factors (Tran et al., 2025). The enzyme methylates and stabilizes key transcriptional regulators, including members of the FoxO family, thereby promoting stress resistance. Sex-specific differences in PRMT7 function have been observed, with female mice showing delayed onset of cardiac pathology following PRMT7 deletion, potentially due to protective effects of estrogen signaling (Ahn et al., 2024).

**Figure 3:**
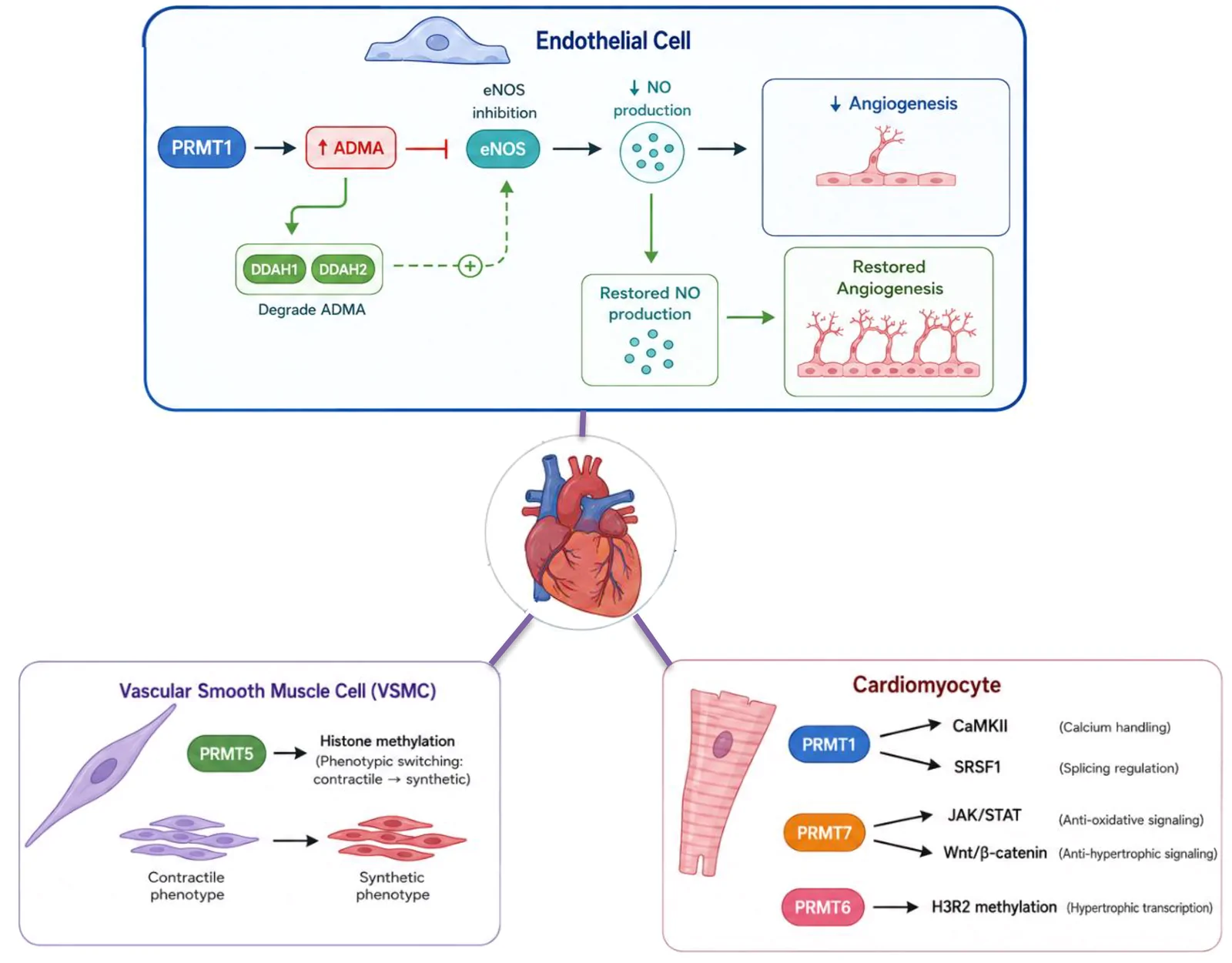
Cell-type specific roles of PRMTs in cardiovascular remodeling. This schematic summarizes the major arginine methyltransferase–dependent pathways in key cardiovascular cell types. In endothelial cells, PRMT1 increases ADMA, which inhibits endothelial nitric oxide synthase (eNOS), leading to reduce NO production and impaired angiogenesis. DDAH1/2 degrade ADMA, thereby restoring NO bioavailability and angiogenic capacity. In vascular smooth muscle cells, PRMT5-mediated histone methylation drives phenotypic switching from a contractile to a synthetic state, contributing to vascular remodeling. In cardiomyocytes, PRMT1 regulates CaMKII-dependent calcium handling and SRSF1-mediated splicing, while PRMT7 modulates JAK/STAT and Wnt/β-catenin signaling to limit oxidative stress and hypertrophic responses. PRMT6 promotes hypertrophic transcription through H3R2 methylation. Together, these cell-type specific PRMT actions integrate epigenetic and signaling mechanisms that shape cardiac and vascular remodeling in cardiovascular disease.

Recent studies demonstrate that PRMT7 regulates the JAK/STAT/SOCS3 signaling axis in cardiomyocytes (Ahn et al., 2024). By promoting SOCS3 expression, PRMT7 provides negative feedback regulation of pro-inflammatory cytokine signaling. This mechanism explains the increased inflammatory responses and adverse remodeling observed in PRMT7-deficient hearts (**Figure 3**).

PRMT2 has been implicated in cholesterol metabolism and atherosclerosis by modulating Liver X Receptor (LXR)-mediated ABCA1 expression. Under diabetic or high-glucose conditions, a reduction in PRMT2 expression impairs macrophage cholesterol efflux, and its deficiency in Prmt2 knockout models is associated with altered cholesterol profiles and accelerated atherosclerotic development (Hussein et al., 2015). In vascular smooth muscle cells, PRMT2 modulates inflammatory responses and proliferation through effects on NF-*κ*B signaling (Zeng et al., 2018).

PRMT6, another type I enzyme, contributes to cardiac hypertrophy through differential regulation of histone methylation. PRMT6 overexpression promotes cardiomyocyte hypertrophy through activation of hypertrophic transcriptional programs, while PRMT6 inhibition attenuates stress-induced remodeling (Raveendran et al., 2020).

PRMT9 has recently been identified as a regulator of macrophage function in myocardial infarction (Bai et al., 2026). Macrophage-specific PRMT9 ameliorates acute myocardial infarction by promoting symmetric dimethylation and subsequent degradation of STAT1, thereby limiting pro-inflammatory macrophage polarization and tissue damage.

CARM1 (PRMT4) regulates cardiomyocyte maturation and developmental gene programs. Disruption of CARM1-mediated arginine methylation impairs the transition from neonatal to adult cardiac gene expression patterns, affecting calcium handling and metabolic adaptation (Garbutt et al., 2024).

### PRMTs in specific cardiovascular pathologies

Atherosclerosis, characterized by chronic inflammation and lipid accumulation in arterial walls, involves multiple PRMT-mediated mechanisms (**Table 1**). Endothelial dysfunction driven by ADMA ac-cumulation represents an early event in atherogenesis (Corradi et al., 2024). The PRMT-DDAH-ADMA axis is dysregulated in atherosclerotic vessels, with increased PRMT expression and decreased DDAH activity contributing to ADMA accumulation (Pope et al., 2009).

**Table 1:** Summary of cell-type specific functions of PRMT isoforms in the cardiovascular system.

Cell type	PRMT isoform	Molecular target/pathway	Functional impact	Ref
Cardiomyocytes	PRMT1	CaMKII methylation (Arg9/275)	Regulates Ca2+ handling prevents heart failure	(Pyun et al., 2018)
Cardiomyocytes	PRMT1	SRSF1 (splicing regulation)	Controls alternative splicing during hypertrophic stress	(Yan et al., 2024)
Cardiomyocytes	PRMT7	JAK/STAT	Protects against oxidative stress and hypertrophy	(Ahn et al., 2024)
Cardiomyocytes	PRMT7	Wnt/β-catenin signaling	Prevents pathological hypertrophy and fibrosis	(Ahn et al., 2022)
Cardiac Fibroblasts	PRMT5	Smad3 methylation/H4R3me2s	Promotes TGF-beta induced fibrosis and remodeling	(Dong et al., 2023)
Endothelial Cells	PRMT7	Inflammation	Prevents endothelial dysfunction and senescence	(Tran et al., 2025)
Macrophages	PRMT9	STAT1 degradation	Limits pro-inflammatory polarization in MI	(Bai et al., 2026)
Cardiomyocytes	PRMT6	H3R2 methylation	Activates hypertrophic transcriptional programs	(Raveendran et al., 2020)

PRMT5 plays a critical role in vascular smooth muscle cell phenotypic switching, a key process in atherosclerotic plaque development and stability. PRMT5 expression increases in synthetic VSMCs and regulates the transition from contractile to proliferative phenotypes through symmetric dimethylation of histones at lineage-specific gene loci. Pharmacological inhibition of PRMT5 reduces neointimal formation in models of vascular injury (Zhu et al., 2023).

Inflammatory processes in atherosclerosis involve PRMT-mediated regulation of immune cell function. PRMT5 regulates macrophage polarization and inflammatory cytokine production, with implications for plaque inflammation and stability (Zhang et al., 2023).The methylarginine derivatives MMA and ADMA also have direct effects on leukocyte adhesion and infiltration.

Hypertension, a major cardiovascular risk factor, involves vascular dysfunction characterized by impaired endothelium-dependent vasodilation (Ma et al., 2023). ADMA contributes to hypertension pathogenesis by reducing nitric oxide-mediated vasodilation and promoting vascular remodeling. Plasma ADMA levels correlate with blood pressure and vascular resistance in hypertensive patients.

PRMT1 and PRMT2 regulate renal sodium handling and vascular tone, contributing to blood pressure homeostasis (Nanayakkara et al., 2005). Angiotensin II, a key mediator of hypertension, upregulates PRMT expression while increasing oxidative stress and DDAH inhibition, creating a feed-forward mechanism that amplifies ADMA accumulation and vascular dysfunction.

Heart failure represents the common end-stage of various cardiovascular diseases, involving com-plex remodeling of cardiac structure and function. Multiple PRMT isoforms contribute to heart failure pathogenesis through distinct mechanisms (Tang et al., 2025).

In dilated cardiomyopathy, decreased PRMT1 activity impairs CaMKII regulation and calcium handling (Pyun et al., 2018). Histone methylation patterns are broadly altered in failing hearts, with changes in both type I and type II PRMT-mediated marks (Szulik et al., 2020).PRMT5-mediated symmetric dimethylation increases at fibrotic gene promoters, contributing to interstitial fibrosis and diastolic dysfunction (Katanasaka et al., 2024).

Metabolic remodeling in heart failure involves PRMT-mediated regulation of gene expression programs controlling energy substrate utilization. PRMTs regulate the expression of genes involved in fatty acid oxidation, glucose metabolism, and mitochondrial function (vanLieshout and Ljubicic, 2019).

Myocardial infarction triggers complex cellular responses involving cardiomyocyte death, inflammation, and subsequent remodeling. PRMTs regulate multiple aspects of the ischemic response. PRMT7 protects against ischemia-reperfusion injury through antioxidant and anti-apoptotic mechanisms (Tran et al., 2025).

PRMT9 modulates macrophage function in the infarcted myocardium, with macrophage-specific PRMT9 ameliorating tissue damage through regulation of STAT1 signaling (Bai et al., 2026). PRMT1 levels decline during ischemia, contributing to increased susceptibility to cell death (Liu et al., 2025).

PRMT1 regulates cardiac ion channel function through methylation of potassium channel subunits. Modulation of the slow activating delayed rectifier potassium current (IKs) by PRMT1 affects action potential duration and may contribute to arrhythmia susceptibility. PRMT-mediated regulation of calcium handling proteins also influences arrhythmogenesis through effects on calcium transient dynamics (An et al., 2022).

### Therapeutic targeting of PRMTs

The recognition of PRMTs as therapeutic targets has driven extensive drug discovery efforts (**Table 2**). PRMT inhibitors can be broadly classified based on their mechanism of action and isoform selectivity (Hu et al., 2016). SAM-competitive inhibitors target the methyl donor binding site, while substrate-competitive inhibitors block arginine recognition. Allosteric inhibitors that bind outside the active site represent an emerging class with potential for improved selectivity.

**Table 2:** Overview of selective PRMT inhibitors and their potential applications in cardiovascular therapeutics.

Compound	Target	Mechanism	Development status	Cardiovascular relevance	Ref
MS023	Type I PRMTs	Substrate-competitive	Preclinical	Reduces vascular inflammation and VSMC switching	(Aparnathi et al., 2025)
GSK3326595	PRMT5	SAM-competitive	Phase Ib	Potential anti-fibrotic agent in heart failure	(Gounder et al., 2025)
GSK3368715	Type I PRMTs	Reversible inhibitor	Phase I (advanced solid tumors)	Potential for reducing systemic ADMA levels	(El-Khoueiry et al., 2023)
SGC707	PRMT3	Allosteric	Preclinical	Regulation of hepatic lipid metabolism	(Kaniskan et al., 2015)

SAM, S-adenosylmethionine; VSMC, vascular smooth muscle cell; ADMA, asymmetric dimethylarginine; eNOS, endothelial nitric oxide synthase.

Type I PRMT inhibitors include DCPR049_12 (compound 28d), a potent inhibitor that has been extensively investigated in leukemia research (Wang et al., 2017). More selective inhibitors such as MS023 show improved potency against type I PRMTs including PRMT1, PRMT3, PRMT4, PRMT6, and PRMT8, binding directly to the substrate binding site (Eram et al., 2016).Structure-guided design has yielded PRMT1-selective inhibitors with reduced off-target effects.

Type II PRMT inhibitors have advanced furthest in clinical development (Li et al., 2019). GSK3326595 is a potent and selective PRMT5 inhibitor that has entered clinical trials for oncology indications (Gounder et al., 2025).Other PRMT5 inhibitors including PF-06939999 are also under clinical investigation. These compounds show favorable pharmacokinetic properties and acceptable safety profiles in early-phase studies (Jensen-Pergakes et al., 2022). While some Type I PRMT inhibitors like GSK3368715 are currently being evaluated in clinical trials for oncology (El-Khoueiry et al., 2023), their specific therapeutic potential and safety profile in cardiovascular pathology remain to be fully elucidated.

Preclinical studies have demonstrated cardiovascular benefits of PRMT modulation in various disease models. PRMT5 inhibition attenuates cardiac fibrosis in models of pressure overload and cardiotoxicity (Katanasaka et al., 2025).The antifibrotic effects are mediated through inhibition of TGF-*β* signaling and reduced fibroblast activation.

Enhancement of PRMT1 activity or expression represents a potential strategy for heart failure treatment. Pharmacological approaches to enhance PRMT1 activity are less developed but represent an active area of investigation (Luo et al., 2025).

ADMA-lowering strategies target the PRMT-DDAH axis at multiple levels. DDAH activators and antioxidants that preserve DDAH activity reduce ADMA accumulation and improve endothelial function (Maas, 2005). Direct ADMA-lowering therapies using recombinant DDAH or ADMA-absorbing compounds are in preclinical development.

L-arginine supplementation represents an established approach to overcome ADMA-mediated NOS inhibition. However, clinical results have been mixed, and concerns about potential pro-atherogenic effects of high-dose arginine have limited widespread adoption (Wilcken et al., 2007).

While no PRMT inhibitors have been approved specifically for cardiovascular indications, several agents are in clinical development. PRMT5 inhibitors initially developed for oncology are being evaluated for potential cardiovascular applications based on their antifibrotic effects (Feustel and Falchook, 2022). The safety profiles established in oncology trials will facilitate repurposing efforts for cardiovascular diseases.

ADMA has been extensively studied as a biomarker for cardiovascular risk stratification (Böger, 2005). Prospective studies have validated ADMA as an independent predictor of cardiovascular events, and ADMA-guided risk stratification may improve patient selection for intensive preventive therapies.

## Future directions and challenges

A major challenge in PRMT therapeutics is achieving isoform selectivity. Given the diverse functions of different PRMT family members, pan-inhibitors may produce unacceptable side effect (Cao et al., 2025). Recent advances in structure-based drug design have begun to address this limitation. High-resolution structural studies, including cryo-EM–guided ligand pose selection and active-site–focused computational modeling, have revealed subtle conformational differences between PRMT catalytic pockets (Zhou et al., 2022). These insights have enabled the rational development of isoform-selective inhibitors, with particularly notable progress for PRMT5 relative to PRMT1, where structure-guided optimization has yielded compounds with improved potency, SAM-competitive behavior, and enhanced biochemical selectivity (Zhou et al., 2022; Chen et al., 2024). These advances in selective inhibitor design complement parallel efforts to develop biomarkers capable of guiding PRMT-targeted therapies.

The development of validated biomarkers is essential for guiding PRMT-targeted therapies. Plasma ADMA serves as a functional readout of PRMT-DDAH pathway activity and may identify patients most likely to benefit from ADMA-lowering interventions (Arrigoni et al., 2010). Novel biomarkers reflecting specific PRMT isoform activities would enable more precise therapeutic targeting.

Imaging approaches to assess myocardial methylarginine metabolism or PRMT activity could provide real-time monitoring of target engagement and therapeutic response. Positron emission tomography tracers targeting the PRMT-DDAH-ADMA axis are in early development (Zakrzewicz et al., 2012).

PRMT-targeted therapies may be most effective when combined with established cardiovascular treatments (Smith et al., 2018). Statins, in particular, have been shown to reduce circulating ADMA levels through mechanisms linked to enhanced DDAH activity. Experimental studies demonstrate that statins can upregulate DDAH1 and DDAH2 transcription via sterol-responsive regulatory pathways, thereby accelerating ADMA degradation and improving endothelial NO bioavailability (Ivashchenko et al., 2010). These mechanistic findings are supported by clinical evidence: an updated systematic review and meta-analysis confirms that statin therapy significantly lowers plasma ADMA concentrations across diverse patient populations, independent of baseline cholesterol levels (Zinellu and Mangoni, 2022). Together, these observations suggest that statins may exert complementary effects when combined with direct PRMT modulators, providing a mechanistic basis for potential additive or synergistic therapeutic interactions.

Genetic variants in PRMT and DDAH genes affect enzyme expression and activity, contributing to individual differences in cardiovascular risk (Hannemann et al., 2022). Pharmacogenomic approaches may identify patients most likely to benefit from PRMT-targeted therapies. Polymorphisms affecting ADMA metabolism have been associated with differential responses to cardiovascular interventions.

## Conclusion

Protein arginine methyltransferases represent a family of enzymes with diverse and critical functions in cardiovascular biology. Through their canonical roles in protein methylation and their indirect effects via ADMA production, PRMTs regulate vascular homeostasis, cardiac remodeling, and responses to ischemic injury. Dysregulation of PRMT activity contributes to the pathogenesis of atherosclerosis, hypertension, heart failure, and other cardiovascular diseases.

The development of PRMT inhibitors has progressed rapidly, with several agents demonstrating efficacy in preclinical cardiovascular models. PRMT5 inhibitors show particular promise for treating cardiac fibrosis, while strategies to enhance PRMT1 activity may benefit patients with heart failure. The PRMT-DDAH-ADMA axis offers multiple intervention points for improving endothelial function and reducing cardiovascular risk.

Future research should focus on developing isoform-selective inhibitors with favorable safety profiles, validating biomarkers for patient stratification, and conducting clinical trials to establish the efficacy of PRMT-targeted therapies in cardiovascular disease. As our understanding of PRMT biology continues to expand, these enzymes are poised to become important therapeutic targets for the treatment of cardiovascular diseases.
